# Supplementary material for: Axitinib Rechallenge Restores the Anticancer Effect after Nivolumab: A Case Report
Source: Int J Mol Sci. 2023 Jul 29;24(15):12149. doi: 10.3390/ijms241512149 (PMC10419223; doi:10.3390/ijms241512149)

Supplementary Table S1: The laboratory findings of the case of cases

Hemogram (202003)				
		Case 1	Case 2	Normal range
WBC	1000/uL	10,3	4.6	3.9-10.6
Hb	g/dL	14.8	15,4	13.5-17.5
Platelets	1000/uL	236	158	150-400
Seg.	%	80.7	64.9	42-74
Biochemistry (202003)				
		Case 1	Case 2	Normal range
Cr.	mg/dL	1.2	1.0	0.64-1.27
ALT	U/L	8	17	10-50
Total Bilirubin	mg/dL	0.5	0.8	≦1.2

Abbreviation: WBC: white blood cell; Hb: hemoglobin; Seg.: Segment; Cr.: Creatine

Supplementary Figure S1: The clinical course of treatment with Case 1 and Case 2 1B: Current image of both patient 1C: The 245 pre- and post-axitnib with Case 1 and Case 2 1D: The dramatic change after axitnib rechallenge with Case 1.

A

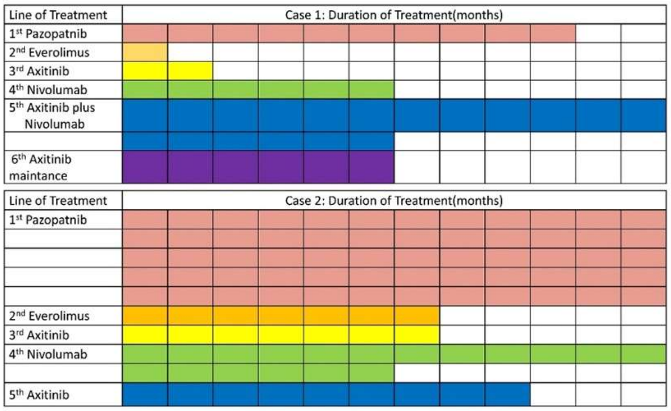

B

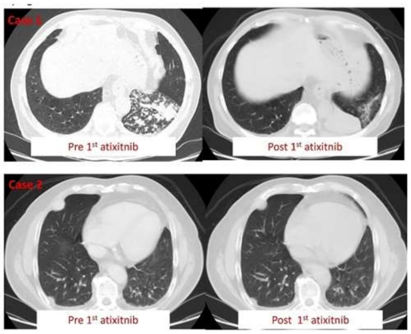

C

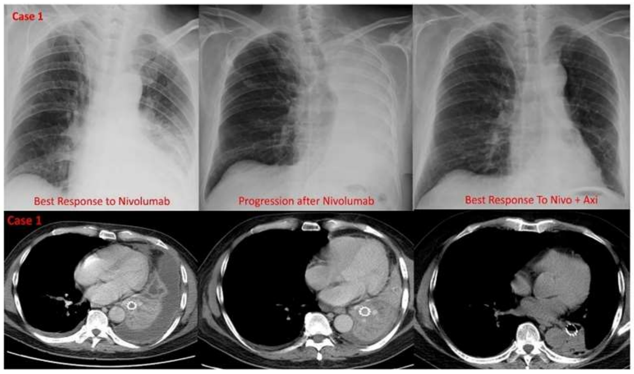

Supplementary Figure S2: The drug sensitivity of programmed death-ligand 1(PD-L1) in the RCC cell line. A. No association was observed between PD-L1 expression and the drug sensitivity of axitinib

A

PD-L1 & axitinib

Drug	Group	Number	Pearson	Spearman	Slope	Intercept	p-value (linregress)
axitinib	Expression	6	-0.353	-0.2	-6.84E-01	3.13E+00	4.93E-01

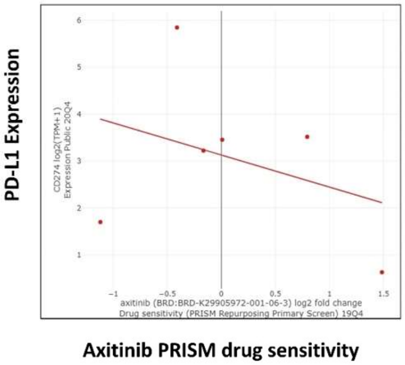

Supplement: Supplementary file 1 [file ijms-24-12149-s001.zip › ijms-2491820-supplementary.pdf]
